# Impact of preexisting proteinuria on the development of regorafenib-induced problematic proteinuria in real-world metastatic colorectal cancer treatment

**DOI:** 10.1038/s41598-024-55727-w

**Published:** 2024-03-02

**Authors:** Yoshitaka Saito, Yoh Takekuma, Yoshito Komatsu, Mitsuru Sugawara

**Affiliations:** 1 Department of Clinical Pharmaceutics & Therapeutics, Faculty of Pharmaceutical Sciences, Hokkaido University of Science, 4-1, Maeda 7-Jo 15-Chome, Teine-Ku, Sapporo, Hokkaido 006-8585 Japan; 2 Department of Pharmacy, Hokkaido University Hospital, Kita 14-Jo, Nishi 5-Chome, Kita-Ku, Sapporo, 060-8648 Japan; 3 Cancer Center, Hokkaido University Hospital, Kita 14-Jo, Nishi 5-Chome, Kita-Ku, Sapporo, 060-8648 Japan; 4 Laboratory of Pharmacokinetics, Faculty of Pharmaceutical Sciences, Hokkaido University, Kita 12-Jo, Nishi 6-Chome, Kita-Ku, Sapporo, 060-0812 Japan

**Keywords:** Regorafenib, Proteinuria, Vascular endothelial growth factor (VEGF), Multikinase inhibitor, Preexisting proteinuria, Risk factor, Gastrointestinal cancer, Oncology

## Abstract

Regorafenib is the first multikinase inhibitor for treating metastatic colorectal cancer (mCRC). Proteinuria is a frequently encountered adverse effect, regardless of prior administration of vascular endothelial growth factor inhibitors. Herein, we aimed to assess the impact of baseline preexisting proteinuria on regorafenib-induced problematic proteinuria during real-world mCRC therapy. Patients with mCRC receiving regorafenib (n = 100) were retrospectively assessed and divided into control and preexisting proteinuria (baseline grade of 1–2) groups. The primary endpoint was the development of grade ≥ 2 (grade ≥ 3 in case of baseline grade 2 patients) proteinuria. Propensity score-matching was performed to confirm the robustness of primary analyses. Defined proteinuria occurred in 30.7 and 57.9% of patients in the control and preexisting proteinuria groups, respectively, with significant differences in the all-patient population (*P* = 0.01). The preexisting proteinuria group exhibited significant defined proteinuria development within 7 days of regorafenib initiation, grade ≥ 3 symptoms, and treatment suspension owing to proteinuria. Similar results were obtained in the propensity score-matched population. According to multivariate logistic regression analysis, baseline proteinuria was a singular risk factor for defined proteinuria development (adjusted odds ratio; 3.76, 95% confidence interval; 1.45–9.75, *P* = 0.007). Collectively, our study revealed that patients with preexisting proteinuria develop regorafenib-induced proteinuria degradation.

## Introduction

Regorafenib is the first small-molecule multikinase inhibitor developed to treat metastatic colorectal cancer (mCRC)^1,2^. However, regorafenib administration is frequently associated with adverse effects such as hand-foot-skin reaction, liver impairment, hypertension, fatigue, anorexia, voice changes, and proteinuria^1,2^.

Globally, proteinuria was found to occur in 8% of regorafenib-treated patients during phase III trial for mCRC therapy^1^. However, a Japanese study has reported proteinuria in 40% of treated patients, including 6% of grade 3 cases^2^. The mechanism underlying regorafenib-induced proteinuria remains poorly understood. However, bevacizumab, a representative vascular endothelial growth factor (VEGF)-targeting monoclonal antibody, is known to induce nephrotoxicity with major histological changes in the glomerulus, resulting in severe defects in the glomerular filtration barrier, which prevents the leakage of serum proteins into the urine^3–8^; this mechanism is considered as a class effect of VEGF-targeting agents^9–11^, which could be applied to regorafenib-induced symptoms. Therefore, it is desirable to clarify the nature and treatment of symptoms to ensure appropriate management.

Risk factors associated with symptoms induced by other anti-VEGF treatments, including multikinase inhibitors and VEGF-targeting monoclonal antibodies, include prolonged administration periods, Asian ethnicity, low baseline glomerular filtration rate (GFR), co-morbidities such as hypertension, diabetes, and hyperlipidemia, prior nephrectomy, calcium channel blocker use, no renin-angiotensin system inhibitor (RASI) use, and baseline proteinuria existence^12–18^. Of these factors, baseline preexisting proteinuria can be problematic, given that anti-VEGF antibodies such as bevacizumab, ramucirumab, and aflibercept beta have already been administered as front-line therapy in mCRC^19^, with approximately 20–60% of patients developing proteinuria^20–22^. Additionally, the above-described studies did not evaluate the population of patients treated with second or later anti-VEGF treatment alone. Considering the duplication of mechanisms of action, VEGF modification by previous treatment can impact the incidence of regorafenib-induced symptoms. Accordingly, we aimed to assess the impact of baseline preexisting proteinuria on regorafenib-induced problematic proteinuria during real-world mCRC therapy.

## Results

### Patient characteristics

**Figure 1:**
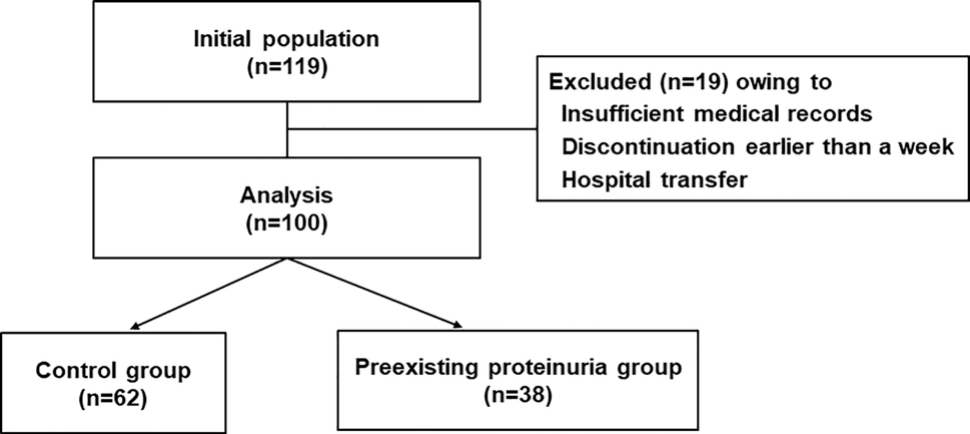
Study design.

In total, 100 patients with mCRC were enrolled in this retrospective observational study (Fig. 1). Table 1 summarizes the baseline patient characteristics. There were no differences between the groups regarding age, Eastern Cooperative Oncology Group performance status (ECOG-PS), primary site, recurrence setting, KRAS status, presence of liver metastasis, body surface area (BSA), body mass index (BMI), hemoglobin, platelet counts, serum albumin levels, liver impairment (grade ≥ 1 aspartate aminotransferase, alanine aminotransferase, total bilirubin elevation), number of prior treatment regimens, duration from the last anti-VEGF treatment administration, hypertension, antihypertensive treatment (except calcium channel blocker use), diabetes mellitus, and starting dosage of regorafenib in all-patient population. The preexisting proteinuria group included males and patients with calcium channel blocker use at a significantly higher rate, with significantly lower estimated GFR (eGFR) than the control group. However, no background difference was confirmed in the propensity score-matched population. In the preexisting group, the baseline severity of proteinuria was 60.5% for grade 1 and 39.5% for grade 2.

**Table 1:** Patient characteristics.

	All-patient population			Propensity score-matched population		
	Control group (n = 62)	Preexisting proteinuria group (n = 38)	*P*-value	Control group (n = 27)	Preexisting proteinuria group (n = 27)	*P*-value
Sex (male/female, n)	29/33	26/12	0.04*	17/10	18/9	1.00
Age (median, range)	64 (28–80)	66 (39–83)	0.25	65 (28–80)	66 (39–83)	0.92
Performance status (ECOG, n) 0–1/2	59/3	35/3	0.67	25/2	25/2	1.00
Primary site (n, %)						
Rectum	25 (40.3%)	11 (29.0%)		13 (48.2%)	7 (25.9%)	
Sigmoid colon	21 (33.9%)	12 (31.6%)		6 (22.2%)	10 (37.0%)	
Ascending colon	4 (6.5%)	9 (23.7%)		2 (7.4%)	5 (18.5%)	
Descending colon	5 (8.1%)	3 (7.9%)		4 (14.8%)	3 (11.1%)	
Cecum	3 (4.8%)	3 (7.9%)		1 (3.7%)	2 (7.4%)	
Transverse colon	3 (4.8%)	0 (0%)		1 (3.7%)	0 (0%)	
Ileocecal region	1 (1.6%)	0 (0%)		0 (0%)	0 (0%)	
Right/left	12/50	12/26	0.23	5/22	7/20	0.74
Recurrence (n, %)	28 (45.2%)	20 (52.6%)	0.54	10 (37.0%)	10 (37.0%)	1.00
KRAS status wild (n, %)	28 (45.2%)	20 (52.6%)	0.54	12 (44.4%)	11 (40.7%)	1.00
Liver metastasis presence (n, %)	41 (66.1%)	25 (65.8%)	1.00	20 (74.1%)	19 (70.4%)	1.00
BSA (m^2^) (median, range)	1.59 (1.36–2.10)	1.65 (1.28–2.06)	0.33	1.69 (1.40–2.10)	1.69 (1.28–2.02)	0.88
BMI (kg/m^2^) (median, range)	22.9 (16.9–30.4)	23.9 (15.0–29.8)	0.28	23.1 (19.0–29.1)	24.2 (15.6–29.8)	0.68
Hb (g/dL) (median, range)	11.5 (8.4–15.1)	11.0 (7.9–15.0)	0.15	11.2 (8.4–15.1)	11.5 (7.9–15.0)	0.88
Plt (× 10^3^/μL) (median, range)	189 (57–451)	218 (90–703)	0.51	183 (82–451)	220 (90–703)	0.40
Albumin (g/dL) (median, range)	3.8 (2.1–4.7)	3.8 (2.8–4.7)	0.54	3.7 (2.1–4.7)	3.7 (2.8–4.5)	0.65
Liver impairment (n, %)	28 (45.2%)	15 (39.5%)	0.68	10 (37.0%)	11 (40.7%)	1.00
eGFR (mL/min/1.73m^2^) (median, range)	81.4 (43.6–148.1)	70.8 (26.0–97.1)	< 0.01**	80.0 (43.6–148.1)	72.0 (26.0–97.1)	0.06
Number of prior treatment regimens (n) 1–2/ 3 or more	15/47	8/30	0.81	7/20	7/20	1.00
Duration from last anti-VEGF administration (days) (median, range)	100 (11–1086)	43 (14–1554)	0.47	35 (11–866)	43 (14–372)	0.71
Hypertension (n, %)	30 (48.4%)	24 (63.2%)	0.21	17 (63.0%)	14 (51.9%)	0.58
Antihypertension treatment details (n, %)						
Number of hypertensive drugs 0/1/2 or more	35/13/14	15/8/15	0.16	12/4/11	14/5/8	0.76
Type of hypertensive drugs						
ARB or ACEI	20 (32.8%)	17 (44.7%)	0.29	10 (37.0%)	9 (33.3%)	1.00
Calcium channel blockers	15 (24.2%)	19 (50.0%)	< 0.01**	12 (44.4%)	11 (40.7%)	1.00
Diuretics	5 (8.1%)	2 (5.3%)	0.71	5 (18.5)	2 (7.4)	0.42
Beta-blockers	2 (3.2%)	3 (7.9%)	0.37	2 (7.4%)	2 (7.4%)	1.00
Diabetes mellitus (n, %)	6 (9.7%)	6 (15.8%)	0.36	4 (14.8%)	5 (18.5%)	1.00
Starting dosage (n, %)						
160 mg	31 (50.0%)	11 (29.0%)	0.06	9 (33.3%)	9 (33.3%)	1.00
≤ 120 mg	31 (50.0%)	27 (71.1%)		18 (66.7%)	18 (66.7%)	

Liver impairment: grade ≥ 1 aspartate aminotransferase, alanine aminotransferase, or total bilirubin elevation.

Types of antihypertensive agents include re-duplication.

*ECOG* Eastern Cooperative Oncology Group, *BSA* body surface area, *BMI* body mass index, *Hb* hemoglobin, *Plt* platelet, *eGFR* estimated glomerular filtration rate, *VEGF* vascular endothelial growth factor, *ARB* angiotensin receptor blockers, *ACEI* angiotensin-converting enzyme inhibitors.

*P < 0.05, **P < 0.01.

### Incidence of proteinuria

**Figure 2:**
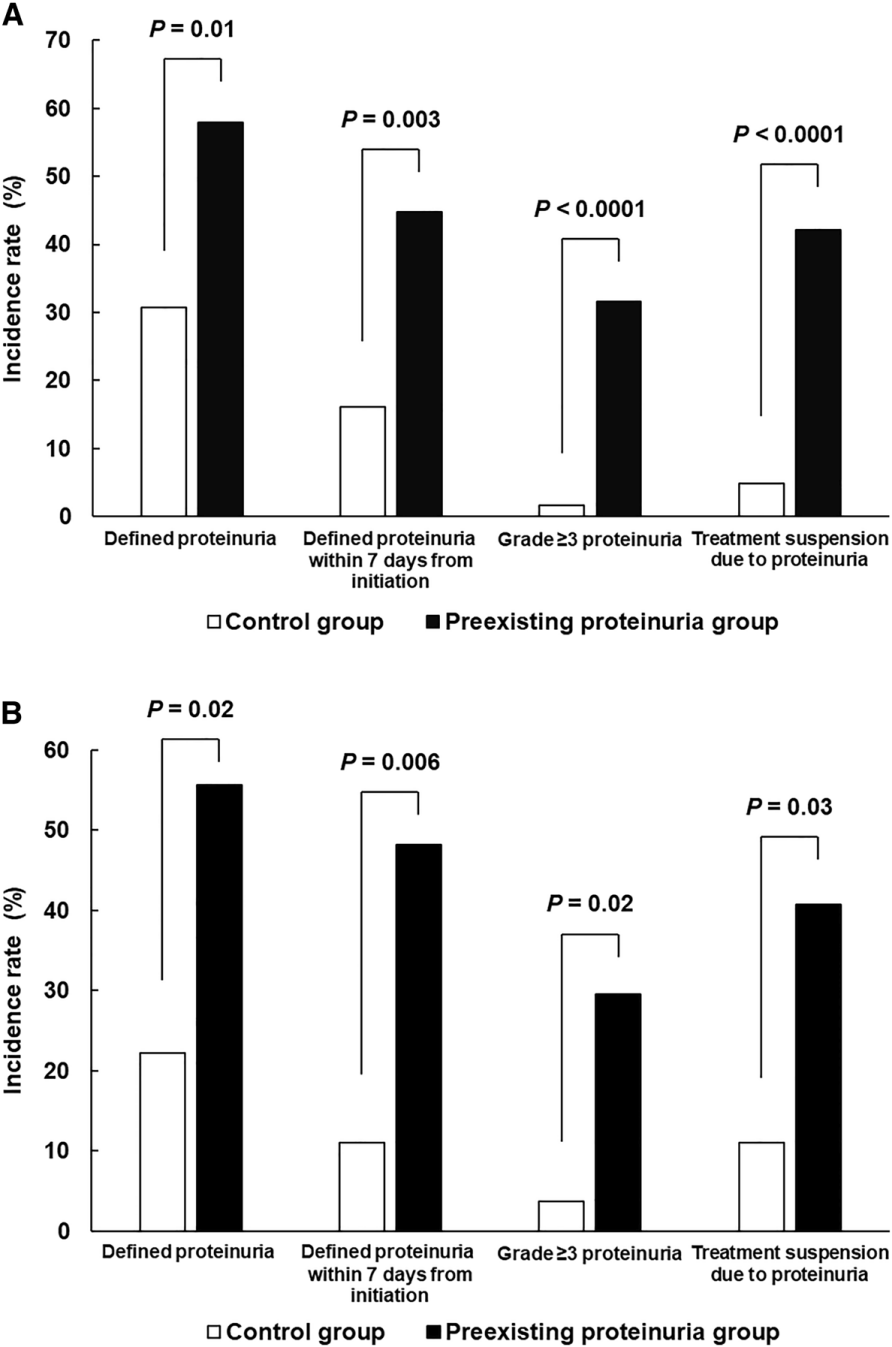
Incidence of defined and grade ≥ 3 proteinuria and treatment suspension due to proteinuria in (**A**) all-patient and (**B**) propensity score-matched populations. In the case of patients with baseline proteinuria of grade 2, symptom development was defined as degradation to grade ≥ 3.

Figure 2 represents data on the incidence and severity of proteinuria during regorafenib therapy. The incidence of defined proteinuria (grade ≥ 2 symptoms for baseline grade 0–1 patients, grade ≥ 3 symptoms in case of baseline grade 2 patients) in the preexisting proteinuria group was 57.9%, which was significantly higher than that in the control group (30.7%) in the all-population analysis, thereby meeting the primary endpoint of the present study (*P* = 0.01, Fig. 2A). In addition, the preexisting group exhibited greater defined proteinuria development within 7 days of regorafenib initiation when compared with the control group (44.7% vs. 16.1%, respectively; *P* = 0.003) (Fig. 2A). The incidence of grade ≥ 3 proteinuria was significantly higher in the preexisting proteinuria group than that in the control group (31.6% vs. 1.6%, respectively; *P* < 0.0001) (Fig. 2A). Furthermore, patients in the preexisting group experienced greater treatment suspension due to proteinuria than those in the control group (42.1% vs. 4.8%, respectively; *P* < 0.0001) (Fig. 2A). These results were also confirmed in the propensity score-matched population (Fig. 2B). The preexisting proteinuria group experienced earlier symptom onset than the control group, although the difference was non-significant (median onset time with range: 7 with 6–35 days in the preexisting group and 11 with 6–140 days in the control group, respectively; *P* = 0.07).

### Risk analysis of defined proteinuria development

Table 2 presents the results of the univariate and multivariate logistic regression analyses to identify risk factors for defined proteinuria development. Baseline preexisting proteinuria was identified as a singular independent risk factor for proteinuria development (adjusted odds ratio: 3.76, 95% confidence interval: 1.45–9.75; *P* = 0.007).

**Table 2:** Univariate and multivariate analyses for risk factors associated with the incidence of regorafenib-induced defined proteinuria.

	Univariate analysis		Multivariate analysis	
	Odds ratio (95% CI)	*P*-value	Odds ratio (95% CI)	*P*-value
Sex Male/female	0.55 (0.25–1.24)	0.15	0.48 (0.17–1.39)	0.18
Age (years) ≥ 65/ < 65	1.62 (0.73–3.62)	0.24	Excluded	–
Performance status (ECOG) 2/0 or 1	0.71 (0.12–4.04)	0.70	Excluded	–
KRAS status Wild/Mutant	1.24 (0.56–2.76)	0.59	Excluded	–
BSA (m^2^) ≥ 1.6/ < 1.6	0.58 (0.26–1.29)	0.178	0.66 (0.24–1.82)	0.42
BMI (kg/m^2^) ≥ 25.0/ < 25.0	1.08 (0.434–2.66)	0.87	Excluded	–
Anemia Present/Absent	1.97 (0.73–5.29)	0.181	Excluded	–
Thrombocytopenia Present/Absent	0.73 (0.30–1.81)	0.50	Excluded	–
Hypoalbuminemia Present/Absent	1.56 (0.62–3.93)	0.34	Excluded	–
Liver impairment Present/Absent	Unable to calculate	–	Excluded	–
Renal impairment Present/Absent	1.56 (0.56–4.35)	0.39	Excluded	–
Treatment line 2nd or 3rd/4th or later	1.44 (0.56–3.67)	0.45	Excluded	–
Dose reduction from treatment initiation Present/Absent	0.96 (0.43–2.16)	0.93	Excluded	–
Duration from last anti-VEGF treatment ≥ 28 days/ < 28 days	1.67 (0.70–3.98)	0.25	Excluded	–
Preexisting diabetes mellitus Present/Absent	2.22 (0.65–7.57)	0.20	Excluded	–
Preexisting hypertension Present/Absent	1.36 (0.61–3.05)	0.45	Excluded	–
Baseline calcium channel blocker use Present/Absent	2.10 (0.91–4.88)	0.08	1.56 (0.63–3.88)	0.34
Baseline RASI use Present/Absent	0.97 (0.42–2.22)	0.94	Excluded	–
Preexisting proteinuria Present/Absent	3.11 (1.34–7.21)	0.008**	3.76 (1.45–9.75)	0.007**

Liver impairment: grade ≥ 1 aspartate aminotransferase, alanine aminotransferase, total bilirubin elevation.

Renal impairment: estimated glomerular filtration rate < 60 mL/min/1.73 m^2^.

*CI* confidence interval, *ECOG* Eastern Cooperative Oncology Group, *BSA* body surface area, *BMI* body mass index, *VEGF* vascular endothelial growth factor, *RASI* renin-angiotensin system inhibitor (angiotensin receptor blocker and angiotensin-converting enzyme inhibitor).

**P < 0.01.

## Discussion

For safe administration of anti-VEGF agents, proteinuria needs to be appropriately managed. It has been reported that patients with mCRC are more likely to develop proteinuria than those with other malignancies^23,24^. Baseline preexisting proteinuria has been associated with symptom exacerbation following treatment with anti-VEGF agents^14–16,18^. However, given that the mCRC treatment strategy differs from that of other malignancies evaluated in previous reports^14–16,18,19^, it is crucial to evaluate the impact of baseline preexisting proteinuria on regorafenib-induced symptoms considering prior anti-VEGF treatment. Thus, we aimed to evaluate the influence of baseline preexisting proteinuria on regorafenib-induced problematic symptoms during real-world mCRC therapy.

Herein, patients with baseline preexisting proteinuria exhibited significantly greater regorafenib-induced proteinuria development than those without baseline symptoms in both all and propensity score-matched populations. In addition, baseline preexisting proteinuria was identified as an independent risk factor for defined proteinuria development. To the best of our knowledge, this is the first report demonstrating increased risk of regorafenib-induced proteinuria in patients with preexisting proteinuria. It can be speculated that baseline symptoms can impact symptom exacerbation during subsequent treatment, although we could not assess whether baseline symptoms could be attributed to prior treatments or primary. Consequently, patients with baseline preexisting proteinuria need to be carefully monitored for ingravescence at every visit.

In contrast, baseline concomitant hypertension and antihypertensive medication, known factors associated with other anti-VEGF treatments, were not identified in the present study^12,13,15–17^. Herein, most symptoms appeared within 7 days from treatment initiation (50.0 and 77.3% in the control and preexisting proteinuria groups, respectively), suggesting that symptom occurs earlier than that with other anti-VEGF treatments for renal cell and hepatocellular carcinoma and lung cancer^13–16^. In our previous evaluation of regorafenib-induced hypertension, the median symptom onset time was also found to be 7 days from treatment initiation^25,26^, suggesting that both proteinuria and hypertension can advance concurrently in regorafenib treatment, possibly due to previous anti-VEGF treatment^25^. Furthermore, previous reports regarding risk factors included anti-VEGF treatment-naïve patients. We consider that these factors substantially contributed to the discrepancies between previous reports and the present study. However, certain patients exhibited symptoms at a later stage. Typically, proteinuria development is closely associated with hypertension, and RASI use has been shown to reduce the risk of development^12,13,15^; hence, the use of RASIs could be a promising treatment strategy for delayed proteinuria development. Further studies are needed to evaluate the association between delayed proteinuria and hypertension and potential management strategies.

There are some limitations to the present study. First, this study was retrospectively conducted at a single institution and included a relatively small number of patients. Second, we evaluated the urine protein/creatinine ratio (UPCR) for substitution to 24-h urine protein evaluation for further diagnosis in case of grade ≥ 2 proteinuria; therefore, the precision of evaluated levels may be debatable. Third, we did not evaluate the impact of proteinuria development on regorafenib treatment efficacy. We have previously reported that the development of regorafenib-induced severe hypertension was substantially associated with improved progression-free survival^26^. As the development of hypertension and proteinuria could be closely associated, considering the mechanisms of development^12,13^, proteinuria may be associated with improved treatment outcomes, although the development of severe proteinuria can limit regorafenib administration. Fourth, we could not evaluate whether baseline proteinuria could be attributed to prior treatments, as some patients transferred from another hospital without sufficient data. Fifth, we included patients with baseline grade 2 proteinuria in the preexisting proteinuria group since we typically administer regorafenib to patients with grade 2 symptoms in clinical practice and attempted to evaluate real world data. Additionally, we assessed the incidence of defined proteinuria in patients with baseline proteinuria of grades 0, 1, and 2, in which were 30.7, 65.2, and 46.7%, respectively. However, as there were less patients with baseline proteinuria, this evaluation is considered insufficient owing to less power. Consequently, further studies assessing its incidence between patients with grade 1 and 2 baseline proteinuria are warranted. Finally, we did not evaluate the blood concentration levels of regorafenib and its metabolites. Reportedly, trough concentration levels of regorafenib and its N-oxide/desmethyl metabolites are closely associated with elevated bilirubin, hypertension, and severe rash, although its association with proteinuria was not evaluated^27^. Assessment of the relationship will contribute to further consideration. Consequently, our preliminary findings should be confirmed in future investigations.

In conclusion, our study revealed that patients with preexisting proteinuria exhibit symptom degradation at a substantially high rate during regorafenib therapy for mCRC. As the symptoms occur earlier than with other anti-VEGF treatments, the establishment of effective management is required.

## Methods

### Patients

The current study enrolled patients with mCRC who received regorafenib between May 2013 and August 2023 at Hokkaido University Hospital. All enrolled patients met the following baseline registry criteria: (1) age ≥ 18 years; (2) 0 to 2 ECOG-PS; (3) baseline proteinuria of grade ≤ 2 (without 3 + proteinuria), (4) sufficient renal or liver function for treatment induction; and (5) adequate medical information available from medical records. Patients who discontinued the treatment earlier than a week after initiation due to disease progression and those transferred to another hospital during the treatment were excluded. The patients were divided into two groups: a control group without baseline proteinuria from May 2013 to May 2022 and a preexisting proteinuria group, including patients with baseline grade 1–2 symptoms from May 2013 to August 2023. We hypothesized that the incidence of defined proteinuria (grade ≥ 2 symptoms for baseline grade 0–1 patients, grade ≥ 3 symptoms in case of baseline grade 2 patients) would be 30% in a control group and 60% in a preexisting proteinuria group, with a patient ratio of 3:2, based on previous reports and our clinical experience^2,14–16,18^. To achieve 80% power with an alpha error of 5%, the required sample size was 60 patients in the control group and 40 patients in the preexisting proteinuria group. Finally, 62 and 38 eligible patients in the control and preexisting groups, respectively, were analyzed.

The study was approved by the Ethical Review Board for Life Science and Medical Research of Hokkaido University Hospital (approval number: 023-0186) and was performed in accordance with the Declaration of Helsinki and the STROBE statement. Given that the present study was retrospectively conducted, informed consent from the participants was waived by the Ethical Review Board for Life Science and Medical Research of Hokkaido University Hospital.

### Treatment methods

Regorafenib at an oral dose of 160 mg/day on days 1–21 was administered every 4 weeks^1,2^; some patients received a reduced starting dose with a subsequent dose-escalation strategy (starting dose of 80–120 mg/day with weekly 40 mg escalation to 160 mg/day if possible), as reported previously^28^. Treatment suspension or dose reduction was performed according to the criteria stated in the medical package insert^29^. Antihypertensive drugs such as calcium channel blockers, RASIs, and diuretic drugs were prescribed at the physician’s discretion to control hypertension.

### Survey of the incidence and severity of proteinuria

All required information was obtained from the patient’s medical records. To assess adverse effects, weekly patient visits were performed during the first 2 months. Laboratory tests, including proteinuria evaluation, were performed at every visit. The severity of adverse effects during the entire treatment period was graded in accordance with the Common Terminology Criteria for Adverse Events version 5.0 and the worst grade of symptom was described. We assessed proteinuria using a simple dipstick test for the first evaluation at every visit, and UPCR assessment instead of 24-h urine protein levels besides dipstick was performed in case of grade ≥ 2 symptom appearance to further evaluate severity, as described previously^14,15,30^.

The primary endpoint was the comparison of defined proteinuria development during all treatment cycles. Secondary endpoints were evaluation of the incidence of defined symptoms within 7 days from treatment initiation and grade ≥ 3 symptoms, symptom onset time, and risk factor(s) associated with defined proteinuria. In addition, propensity score-matching was performed to adjust patients’ factors between the two groups, and matched data were additionally evaluated to verify the robustness of all-patient population results.

### Statistical analysis

The differences in patient characteristics between the control and preexisting proteinuria groups were evaluated using Fisher’s exact probability test for the categorical variables and the Mann–Whitney *U* test for the continuous variables. The incidence of proteinuria was assessed using Fisher’s exact probability method. The symptom onset time was compared using the Mann–Whitney *U* test. Univariate and multivariate logistic regression analyses were performed to identify the independent risk factor(s) associated with the development of defined proteinuria using the following possible baseline covariates: sex, age, ECOG-PS, KRAS status, BSA, BMI, anemia, thrombocytopenia, hypoalbuminemia, liver impairment, renal impairment (eGFR calculated using the formula developed by the Japanese Society of Nephrology^31^, less than 60 mL/min/1.73 m^2^), treatment line, dose reduction from treatment initiation, duration from the last anti-VEGF treatment, preexisting diabetes mellitus and hypertension, calcium channel blocker and RASI use, and preexisting proteinuria based on previous reports^12–18^. Candidate variables with *P* < 0.20 in univariate analysis were sorted in *P*-value ascending order and included in the multivariate analysis (one candidate per 10 events). Propensity score-matching was performed using the following baseline variables: age, sex, serum albumin levels, liver impairment, renal impairment, co-morbidity of hypertension and diabetes mellitus, use of calcium channel blockers and RASIs, and starting dosage. To decrease bias with these potential confounding factors, 1:1 matching (without replacement) in the two groups was carried out using the nearest neighbor method, with a 0.20-width caliper of the standard deviation of the logit of propensity scores.

All analyses were performed using JMP version 16.1 statistical software (SAS Institute Japan, Tokyo, Japan). Differences were considered statistically significant when *P*-values were < 0.05.

### Ethical approval

All the procedures performed in studies involving human participants were carried out in accordance with the ethical standards of the institutional and/or national research committee and with the 1964 Helsinki Declaration and its later amendments or comparable ethical standards. For this type of study, formal consent was waived by the Ethical Review Board for Life Science and Medical Research of the Hokkaido University Hospital.

## Data Availability

The datasets used and/or analyzed during the current study are available from the corresponding author upon reasonable request.
